# Mono-ADP-Ribosylhydrolase MACROD2 Is Dispensable for Murine Responses to Metabolic and Genotoxic Insults

**DOI:** 10.3389/fgene.2018.00654

**Published:** 2018-12-12

**Authors:** Oriana Lo Re, Tommaso Mazza, Manlio Vinciguerra

**Affiliations:** ^1^International Clinical Research Center, St Anne’s University Hospital, Brno, Czechia; ^2^Department of Biology, Faculty of Medicine, Masaryk University, Brno, Czechia; ^3^Bioinformatics Unit, Casa Sollievo della Sofferenza (IRCCS), San Giovanni Rotondo, Italy; ^4^Institute for Liver and Digestive Health, Division of Medicine, University College London, London, United Kingdom

**Keywords:** metabolic stress, obesity, MACROD2, irradiation, genotoxic stress response, knock out mouse model

## Abstract

ADP-ribosylation is an important post-translational protein modification that regulates diverse biological processes, controlled by dedicated transferases, and hydrolases. Disruption in the gene encoding for MACROD2, a mono-ADP-ribosylhydrolase, has been associated to the Kabuki syndrome, a pediatric congenital disorder characterized by facial anomalies, and mental retardation. Non-coding and structural mutations/variations in MACROD2 have been associated to psychiatric disorders, to obesity, and to cancer. Mechanistically, it has been recently shown that frequent deletions of the MACROD2 alter DNA repair and sensitivity to DNA damage, resulting in chromosome instability, and colorectal tumorigenesis. Whether MACROD2 deletion sensitizes the organism to metabolic and tumorigenic stressors, in absence of other genetic drivers, is unclear. As MACROD2 is ubiquitously expressed in mice, here we generated constitutively whole-body knock-out mice for MACROD2, starting from mouse embryonic stem (ES) cells deleted for the gene using the VelociGene^®^ technology, belonging to the Knockout Mouse Project (KOMP) repository, a NIH initiative. MACROD2 knock-out mice were viable and healthy, indistinguishable from wild type littermates. High-fat diet administration induced obesity, and glucose/insulin intolerance in mice independent of MACROD2 gene deletion. Moreover, sub-lethal irradiation did not indicate a survival or lethality bias in MACROD2 knock-out mice compared to wild type littermates. Altogether, our data point against a sufficient role of MACROD2 deletion in aggravating high-fat induced obesity and DNA damage-associated lethality, in absence of other genetic drivers.

## Introduction

ADP-ribosylation, the addition of one or more ADP-ribose moieties from nicotinamide adenine dinucleotide (NAD+) to a protein or a target molecule, was first described in the early 1960’s. ADP ribosylation is a reversible post-translational modification regulating critical cellular pathways in eukaryotes, such as DNA repair and apoptosis, and underlies as well the pathogenicity of certain bacteria (Belenky et al., 2007; Cohen and Chang, 2018). Improper ADP-ribosylation has been implicated in metabolic, inflammatory diseases and several cancers (Bai, 2015). ADP-ribosylation levels are defined by the activities of specific transferases and hydrolases. ADP-ribosylation can be catalyzed by poly(ADP-ribose) polymerases (PARPs), which transfer ADP-ribose chains, or by mono(ADP-ribose) polymerase that add single ADP-ribose blocks. MACROD2 is of one of the tree monoADP-ribosylases (MARs) in humans, together with macroD1 and C6orf130, which possesses reversible ADP-ribosyl hydrolase activity and is required for its recruitment to DNA lesions induced by laser microirradiation (Jankevicius et al., 2013; Rosenthal et al., 2013; Barkauskaite et al., 2015). Abnormalities in the sequence of the gene locus of MACROD2, most often deletions or single nucleotide polymorphisms (SNPs), have been consistently associated with cancers, neurological and psychiatric disorders (Anney et al., 2010; Lionel et al., 2011; Perlis et al., 2012; Cheng et al., 2013; Jahanshad et al., 2013; Linnebacher et al., 2013; Tsang et al., 2013; Jones et al., 2014; Mohseni et al., 2014; Briffa et al., 2015; van den Broek et al., 2015; Hu et al., 2016; Sakthianandeswaren et al., 2018). Beyond the *in vitro* and clinical association studies discussed above, the *in vivo* function and the exact molecular functions of MACROD2 are poorly understood.

A study by Chang et al. (2018) aiming at identifying the genetic loci for circulating VAP-1 levels (Vascular adhesion protein-1, a membrane-bound amine oxidase highly expressed in mature adipocytes and released into the circulation) in 1,100 Han Chinese individuals from 398 families, demonstrated a strong association with MACROD2; siRNA-mediated knockdown of MACROD2 significantly suppressed the expression of key adipogenic genes FABP4, ADIPOQ, CEBPA, PPARG2, and SREBP1 in primary human pre-adipocytes isolated from the visceral adipose tissue, thus maintaining them in an undifferentiated state. Therefore MACROD2 could act as a transcriptional regulator of adipogenesis and obesity, in turn a major metabolic risk factor for developing cancer. In this respect, a recent study demonstrated how MACROD2 haploinsufficiency alters DNA repair and sensitivity to DNA damage, and results in chromosome instability in almost 1/3 of colorectal patients (Sakthianandeswaren et al., 2018). Whereas previous studies investigating the role of MACROD2 on cancer development used nude mice xenograft with human cells harboring altered expression of MACROD2 (Mohseni et al., 2014), the latter was the first study reporting the phenotype of MACROD2 knock-out mice [obtained from the Knockout Mouse Project Repository (KOMP), Jackson Laboratory], which developed normally into adulthood, and then crossed with the ApcMin/+ mouse model. ApcMin/+ mice harbor a truncating germline mutation in Apc, and intestinal tumors arise spontaneously from loss of heterozygosity of the wild-type Apc allele, a mechanism found in human colorectal cancer. Heterozygous and homozygous depletion of MACROD2 enhanced significantly intestinal tumorigenesis in this susceptible genetic background (Sakthianandeswaren et al., 2018). Accordingly, at the clinical level, low nuclear expression of MACROD2 is associated with poor prognosis of patients with stage III primary colon cancer (van den Broek et al., 2018).

Despite these evidences, whether MACROD2 deletion sensitizes the organism to metabolic and tumorigenic stressors, in absence of other genetic drivers, is unclear. Here we generated constitutively whole-body knock-out mice for MACROD2. MACROD2 knock-out mice were viable and healthy, indistinguishable from wild type littermates. High-fat diet administration induced obesity, and glucose/insulin intolerance in mice independent of MACROD2 gene deletion. Moreover, sub-lethal irradiation did not indicate a survival or lethality bias in MACROD2 knock-out mice compared to wild type littermates. Altogether, our data point to a dispensable role for MACROD2 deletion in aggravating the effects of metabolic and tumorigenic stressors, in absence of other genetic drivers.

## Materials and Methods

### MACROD2 Knock-Out Mice Generation

**FIGURE 1:**
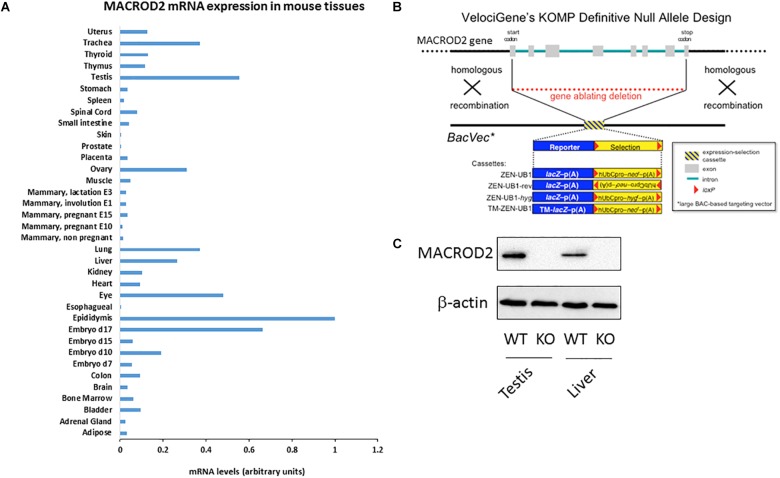
Please provide promoter the meaning of “^∗^” specified in Figure 1. **(A)** qPCR screening of MACROD2 mRNA expression levels normalized to GAPDH across 36 murine somatic and reproductive organs and tissues. **(B)** Generation of MACROD2 mice. Design diagram of VelociGene^®^ KOMP constitutive MACROD2 deletion. LacZ, β-galactosidase; TM-lacZ, transmembrane sequence fused to β-galactosidase; neo or hyg, coding sequences for neomycin or hygromycin phosphotransferases; hUbCpro, promoter from the human ubiquitin C gene; p(A), polyadenylation signal. **(C)** Immunoblot analysis. Protein extracts (10 μg/lane) of liver (L) and kidney (K) were prepared from wild-type (WT) and macroD2 KO (KO) mice and analyzed using anti-macroD2 and anti-β-actin. ^∗^BacVec = BAC based targeting vector.

MACROD2 knock-out (KO) in VGB6 embryonic stem (ES) cells were purchased from the University of California (UC) Davis KOMP: http://velocigene.com/komp/detail/12650, a trans National Institute of Health (NIH) initiative aiming at generating a comprehensive and public resource comprised of mouse ES cells containing a null mutation in every gene in the mouse genome (www.komp.org). Constitutive KO of MACROD2, located on chromosome 2, was achieved using the VelociGene^®^ technology (Valenzuela et al., 2003), according to the design illustrated in Figure 1B. The genotype strategy is illustrated in Supplemental Figure 1. Accordingly, the genotyping primer sequences (5’-3’) were as it follows:

SU, TTCCTGAGCTCCGTGAATG, SD, TCTTTCAAGCTGACTGTGGG;
LacInR, TTGACTGTAGCGGCTGATGTTG, LacInf, GGTAAACTGGCTCGGATTAGGG;
NeoInR, TACTTTCTCGGCAGGAGCAAGGTG, NeoInF, TTCGGCTATGACTGGGCACAACAG;
LacInZRev, GTCTGTCCTAGCTTCCTCACTG, NeoFwd, TCATTCTCAGTATTGTTTTGCC.

Targeted VGB6 ES cells were then injected into C57BL/6 eight cell-stage embryos. Mice heterozygous for the macroD2 allele were further crossed between each other to generate KO mice. All mice used were on a C57Bl/6 genetic background, and were bred and maintained at the EMBL Mouse Biology Unit, Monterotondo, or at Plaisant Srl (Rome, Italy), in accordance with current Italian legislation (article 9, 27 January 1992, number 116) under license from the Italian Health Ministry. EchoMRI quantitative magnetic resonance and CT scan were performed as previously described (Pazienza et al., 2016).

### Irradiation

For survival experiments, total body irradiation was administered to wild type macroH2A1 heterozygous and macroH2A1 KO mice (*n* = 25–30 per group), restrained in holders, using a MK-1-68A Cesium-137 Gamma animal irradiator (J.L. Shepherd and Associates), with 1000 rad in a single dose. After irradiation all animals were returned to the animal facility.

### qPCR

MACROD2 mRNA expression screen was performed on a cDNA library (96 wells coated plates, normalized against GAPDH) from ORIGENE (MNRT101) – TissueScan Mouse Normal Tissue qPCR Array, according to manufacturer’s instructions. The array included cDNA from Adipose Tissue, Adrenal Gland, Bladder, Bone Marrow, Brain, Colon, Embryo d7, Embryo d10, Embryo d15, Embryo d17, Epididymis, Esophagus, Eye, Heart, Kidney, Liver, Lung, Mammary gland (not pregnant), Mammary gland (pregnant E10), Mammary gland (pregnant E15), Mammary gland (involution E1), Mammary gland (lactation E3), Muscle, Ovary, Placenta, Prostate, Skin, Small intestine, Spinal Cord, Spleen, Stomach, Testis, Thymus, Thyroid, Trachea, and Uterus. Real Time-PCR was performed in triplicate utilizing StepOnePlus^TM^ Real-Time PCR System (Applied Biosystems, Darmstadt, Germany) and SYBRTM Select Master Mix (ThermoScientific). Primer sequences were as it follows (5’-3’): MACROD2: sense, GCCTGAGACGGTTATGGAAA; antisense, TGTCTCCCACCCTTCTTGTC. 18S: sense: AGTCCCTGCCCTTTGTACACA; antisense: CGATCCGAGGGCCTCACTA. GAPDH: sense: CGTCCCGTAGACAAAATGGT; antisense: TCAATGAAGGGGTCGTTGAT. Fold changes between groups were calculated using the 2^-ΔΔct^ method.

### Immunoblotting

Protein extraction and immunoblotting analyses were performed as previously described (Benegiamo et al., 2012; Borghesan et al., 2016). Primary antibodies were as follows: anti-macroD2 (ThermoScientific, Cat. PA5-45950), anti-β-actin (Cell Signaling, Cat. 4967).

### Comet Assay

Mouse embryonic fibroblasts (MEFs) were isolated as previously described (Di Biase et al., 2017) from day 10.5 gestation embryos, washed in phosphate-buffered saline, and disaggregated with an 18-gauge needle. Following three washes in Dulbecco’s modified Eagle medium (DMEM), the suspension was plated in a 60-mm dish in DMEM containing 15% fetal calf serum. MEF were isolated at 37°C and 5% CO2 in either 3 or 21% oxygen. After 24 h cells were trypsinized and replated to enrich for fibroblasts in DMEM containing 10% fetal calf serum. Until the genotypes (WT or MACROD2 KO) were confirmed, each embryo was cultured separately. After 2 days, cells with an identical genotype were pooled and designated passage 1. A total of 10^5^ cells were reseeded at each subculture. MEFs were not passaged for more than 2 times, kept on ice and subjected to 10 Gy IR, and were then harvested immediately for Comet Assay. Comet Assay on MEFs was performed using the CometAssay^®^ Kit (25 × 2 well slides) (Trevigen, BioTechne ltd, United Kingdom), according to manufacturer’s instructions. Briefly, the Comet Assay kit was used to perform alkali denaturing comet assays. Samples were stained with SYBR green prior to analysis by fluorescence microscopy. Pictures of individual cells were taken with a Zeiss AxioObserver Z1 inverted microscope equipped with a black-and-white CCD camera. The percentage of tail DNA was analyzed from 50 cells per sample using CaspLab software.

### Glucose Tolerance Test (GTT) and Insulin Tolerance Test (ITT)

GTT and ITT were performed as previously described (Sheedfar et al., 2015; Pazienza et al., 2016). Briefly, Mice were fasted for 6 h during the daytime, and given intraperitoneally (IP) injection with glucose solution or with human recombinant insulin (0.5 U kg – 1 body weight, Actrapid; Novo Nordisk[insulin tolerance test (ITT)]. Blood was collected from the tail vein and glucose levels were measured with an OneTouch Ultra glucometer (Lifescan Benelux, Belgium) before and 30, 60, and 120 after the gavage/injection.

### Bioinformatic Analysis of MACROD2 SNPs in Human Cancers

Single Nucleotide Polymorphisms (SNPs) and short insertions/deletions (indels) were sought in an ad-hoc database from Wellcome Sanger Institute (i.e., COSMIC ver. 86). COSMIC includes genomic data of the following tissues: Adrenal gland, Autonomic ganglia, Biliary tract, Bone, Breast, Central nervous system, Cervix, Endometrium, Eye, Fallopian tube, Gastrointestinal tract (site indeterminate), Genital tract, Haematopoietic and lymphoid, Kidney, Large intestine, Liver, Lung, Meninges, NS, esophagus, Ovary, Pancreas, Parathyroid, Pituitary, Placenta, Pleura, Prostate, Salivary gland, Skin, Small intestine, Soft tissue, Stomach, Testis, Thymus, Thyroid, Upper aerodigestive tract, Urinary tract, and Vulva. Information on the chromosome, start and end positions of each genomic variant, together with reference and alternate alleles were downloaded from COSMIC and given in input to ANNOVAR^1^. ANNOVAR was used to retrieve information on the genomic regions hit by each variant (i.e., exon, intergenic, splicing, introns, non-coding RNA genes, UTRs), and to predict their functional consequences (i.e., non-synonymous SNV, synonymous SNV, frameshift insertion, frameshift deletion, non-frameshift insertion, non-frameshift deletion, frameshift block substitution, non-frameshift block substitution). Frequency of genomic regions and mutation types were plotted in R ver. 3.5.0 as pie-charts, using the ggplot2 package.

### Statistical Methods

Data are shown as means ± standard error of the mean (SEM). Groups were compared with either Student’s *t*-test or the non-parametric Mann–Whitney U-test, as appropriate, using GraphPad Prism Software (version 5.00 for Windows, San Diego, CA, United States): significance was *P* ≤ 0.05. Survival analyses of mice employed the Kaplan-Meier estimator.

## Results

### Tissue Distribution of MACROD2 mRNA and Generation of MACROD2 Knock-Out (KO) Mice

A comprehensive characterization of differential MACROD2 transcript expression in different tissues has not been performed. To this purpose, we probed a Mouse Normal Tissue qPCR Array containing first strand DNA from 36 tissues [adipose tissue, adrenal gland, bladder, bone marrow, brain, colon, embryo day7, embryo day 10, embryo day 15, embryo day 17, epididymis, esophagus, eye, heart, kidney, liver, lung, mammary gland (not pregnant), mammary gland (pregnant – 10 days from copulation plug date), mammary gland (pregnant – 15 days from copulation plug date), mammary gland (involution day 1), mammary gland (lactation day 3), muscle, ovary, placenta, prostate, skin, small intestine, spinal cord, spleen, stomach, testis, thymus, thyroid, trachea, uterus] for MACROD2 mRNA expression using specific primers (Figure 1A). High mRNA expression for MACROD2 was detected in the reproductive organs: the epididymis, the testis, the ovary; high MACROD2 expression levels were also detected in the late mouse embryo at day 17, as well as in the lung, in the trachea, in the eye and in the liver (Figure 1A). Of note, MACROD2 mRNA expression was ubiquitous as it was detected in all the 36 tissues tested (Figure 1A). To study the systemic effects of MACROD2 deletion, we endeavored the generation of constitutive MACROD2 knock-out (KO) mice. We obtained ES cells from the knock out mouse project (KOMP) NIH repository, where deletion of MACROD2 GENE was achieved using the VelociGene^®^ technology (Valenzuela et al., 2003), according to the design illustrated in Figure 1B. VelociGene^®^ technology uses a MACROD2 targeting vector based on bacterial artificial chromosomes (BACs), and it allowed to replace the MACROD2 with a lacZ (β-galactosidase) reporter to localize gene expression (Valenzuela et al., 2003; Figure 1B). MACROD2 could be easily detected in wild type (WT) mice and in protein extracts of organs such as the testis and liver by immunoblot analysis, while it was absent in KO mice, validating the MACROD2 genetic deletion strategy (Figure 1C).

### MACROD2 Deletion in Mice Does Not Aggravate Irradiation Induced Lethality and DNA Damage

**FIGURE 2:**
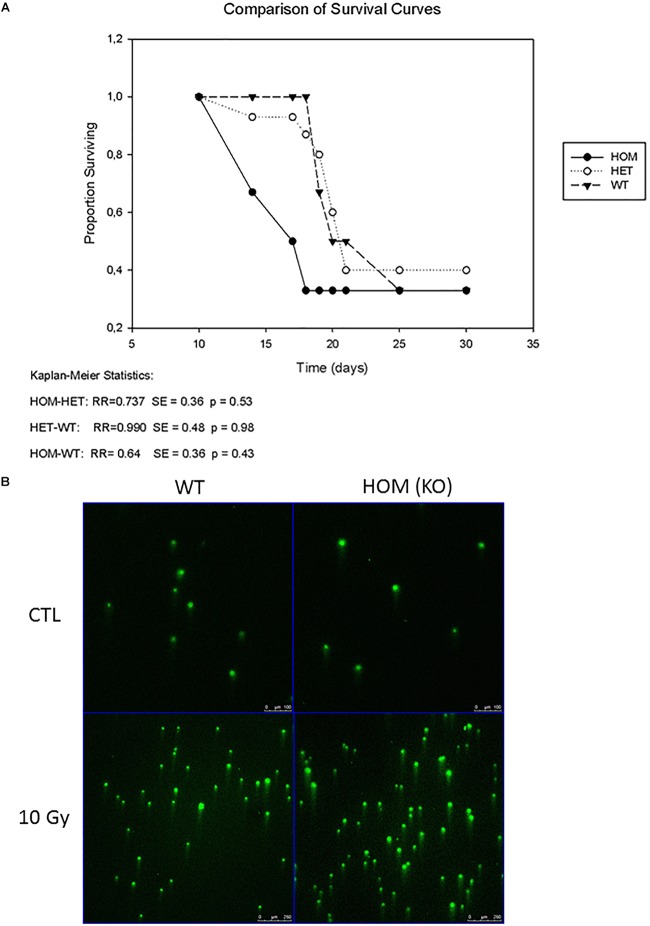
MACROD2 deletion has no effect on γ-irradiation induced lethality and DNA damage. **(A)** A Kaplan–Meier survival curve of wild type (WT), MACROD2 HET (+/-) and MACROD2 KO (HOM; -/-) mice (*n* = 25–30 mice/group). Survivals of mice were closely monitored several times per day. **(B)** Comet Assay. Images of comet assay showing DNA damage mouse fibroblasts in response to γ-irradiation. The cells were processed for comet assays immediately after the irradiation.

ADP-ribosylation is a dynamic post-translation modification that regulates the early phase of DNA repair pathways by recruiting repair factors to chromatin. As ADP-ribosylation can reveerted by the hydrolase activity of MACROD2, it has been directly involved in DNA repair (Golia et al., 2017). To replicate conditions of high levels of DNA damage, we irradiated three cohorts of mice; wild type (controls) (*n* = 30), MACROD2 heterozygous (HET) (*n* = 27) and MACROD2 KO (HOM) (*n* = 25) with a dose of 1000 rad to the whole body. No significant changes in survival rates between group comparisons were observed (Figure 2A). Thus mice lacking MACROD2 do not succumb more easily to the DNA-damaging effects of lethal irradiation. To examine if the wild type, MACROD2 HET and MACROD2 KO fibroblasts experienced the same physical DNA damage in response to γ-irradiation, we subjected cells to 10 Gy of γ-irradiation and collected immediately for comet assay. All three types of cells had similarly increased tail DNA in response to γ-irradiation (Figure 2B), suggesting that they sustain similar levels of DNA damage. Taken together, these *in vivo* and *in vitro* results show that MACROD2 deletion does not worsen DNA damage in response to γ-irradiation.

### MACROD2 Deletion in Mice Does Not Impact High Fat-Diet Induced Obesity, Insulin and Glucose Intolerance

**FIGURE 3:**
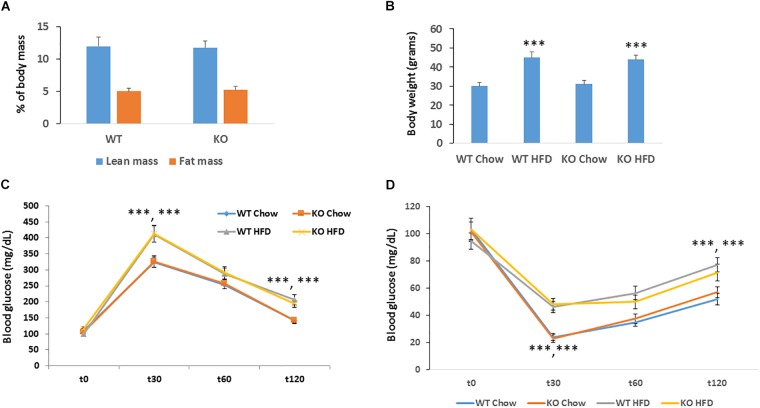
Figure MACROD2 deletion has no effect on high-fat diet induced dysmetabolism. **(A)** lean and fat masses were determined by CT scan in WT and MACROD2 KO mice fed a HF diet (HFD). **(B)** body weight in WT and MACROD2 KO mice fed a chow or a HF diet at the experimental end point. **(C,D)** GTT and ITT were performed in WT and Tg mice fed a chow or a HFD following a 6 h fast. Mice were injected with 2 g glucose/kg of body weight intraperitoneally, and blood glucose concentrations were measured at the indicated time points (minutes). Data are expressed as means ± S.E.M. (*n* = 8–9 per group). ^∗∗∗^*p* < 0.001 change versus WT fed a chow diet.

Obesity is a major risk factor for developing cancer. Since silencing of MACROD2 significantly suppressed the expression of adipogenic genes in primary human pre-adipocytes (Chang et al., 2018), we hypothesized that MACROD2 could play a role in the development of obesity *in vivo*. We thus established a classical model of high fat diet-induced obesity (Pazienza et al., 2016). Upon feeding an obesogenic[12 weeks, 60% energy from lard (Sheedfar et al., 2015)] high fat (HF) diet, MACROD2 KO mice did not show any change in fat induced-increased adiposity as assessed by quantitative EchoMRI/CT scan (Figure 3A). Accordingly, body weight of age-matched MACROD2 KO mice did not differ than wild-type mice both under a chow diet and under a HF diet (Figure 3B). In comparison with wild-type mice, no gross changes in HF diet-induced obesity are observed in MACROD2 KO mice. Nonetheless, we sought to investigate the ability of KO mice to respond to a glucose or insulin challenge. Basal glucose levels were similar in KO versus wild-type mice, fed a chow or a HF diet (Figures 3C,D). In glucose tolerance tests (GTT), glucose levels remained similar in MACROD2 KO mice at every time point, compared to wild-type littermates, both upon a chow or a HF diet (Figure 3C). Insulin tolerance tests (ITT) showed that the insulin-mediated decrease in glycemia was also comparable in MACROD2 KO mice versus wild-type mice at every time measured upon a chow diet (Figure 3D). Altogether these data demonstrate that whole body deletion of MACROD2 gene is irrelevant for the insurgence and progression of obesity, and for its associated dysmetabolism.

## Discussion

MACROD2 is a monoADP-ribosylase that in human cells *in vitro* has been mechanistically shown to be instrumental in DNA stability and tumorigenesis (Jankevicius et al., 2013; Rosenthal et al., 2013; Barkauskaite et al., 2015; Golia et al., 2017; Sakthianandeswaren et al., 2018) and adipogenesis (Mohseni et al., 2014; Chang et al., 2018). MACROD2 has been recently shown to be exported from the nucleus upon DNA damage, in ATM activity-dependent manner, in human U2OS cells (Golia et al., 2017). This nuclear export restricts the time that MACROD2 spend at the DNA lesions, which may decrease the net ADP-ribosylhydrolase activity at these sites of damage (Golia et al., 2017). Low expression of MACROD2 is associated with poor prognosis of colorectal cancer patients (van den Broek et al., 2018). A recent breakthrough report documented the generation of MACROD2 knock-out mice (obtained from the KOMP, Jackson Laboratory), which developed normally into adulthood and displayed significantly enhanced intestinal tumorigenesis in the ApcMin/+ susceptible genetic background (Sakthianandeswaren et al., 2018). In the latter study a phenotyping of MACROD2 KO mice was performed at the histological level in animals at 5 months of age, showing no abnormalities (Sakthianandeswaren et al., 2018). In fact, only in carriers of APC inactivating mutations MACROD2 had epistasis effects enhancing the growth of gastrointestinal tumors (Sakthianandeswaren et al., 2018). The generalization of these data pointing at MACROD2 as a tumor suppressor is thus unclear. In our study, using a newly generated mouse model we elucidated for the first time if whole body MACROD2 deletion alone is sufficient to aggravate the deleterious phenotype triggered by DNA damaging – induced by sub-lethal g-irradiation – and by metabolic stressors – obesogenic high fat diet. Our data show convincingly that MACROD2 deletion alone is dispensable in these processes. RNA and protein analyses confirmed efficient deletion of whole MACROD2 transcript and protein by the VelociGene^®^ technology in multiple tissues examined. The MACROD2 KO mice we generated were viable and healthy, indistinguishable from wild type littermates, consistent with the study of Sakthianandeswaren et al., 2018). These authors acquired MACROD2 KO mice from the Jackson Laboratories (Maine, US), which in turn generated them from the targeted VGB6 ES cells originating from KOMP repository. We used the same cells and the same repository for the in house generation of our mice.

**FIGURE 4:**
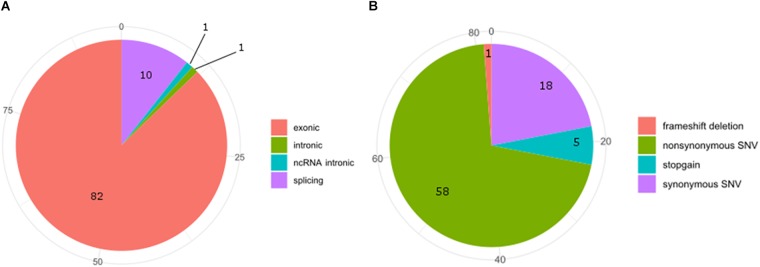
Pie-charts of frequencies of the interested genomic regions **(A)** and functional consequences **(B)** of COSMIC variants hitting the MACROD2 genes.

Exon 5 of MACROD2 gene was originally found disrupted in Japanese children affected by the Kabuki syndrome, a rare, clinically congenital mental retardation syndrome of unknown etiology, characterized by facial anomalies and mental retardation (Maas et al., 2007; Kuniba et al., 2008). Deletions or SNPs in the gene locus of MACROD2 have been often associated with tumor progression, neurological and psychiatric disorders (Anney et al., 2010; Lionel et al., 2011; Perlis et al., 2012; Cheng et al., 2013; Jahanshad et al., 2013; Linnebacher et al., 2013; Tsang et al., 2013; Jones et al., 2014; Mohseni et al., 2014; Briffa et al., 2015; van den Broek et al., 2015; Hu et al., 2016; Sakthianandeswaren et al., 2018). However, in the case of neurological and psychiatric disorders, such as for autism spectrum disorder (ASD) or for attention deficit hyperactive disorder (ADHD), the MACROD2 association failed to replicate in well-powered cohorts (Bradley et al., 2010; Chen et al., 2015). In fact, although it is known that SNPs in regulatory elements residing within intronic regions can alter silencing, enhancer, or splicing events (Chorev and Carmel, 2012; Lee et al., 2012), further work by *in silico* or multi-array analysis of all known SNPs and mutations in the MACROD2 gene region is required to narrow down on the precise mechanisms responsible for the observed phenotypic effects. For instance, SNPs within intron one of several genes have been shown to influence gene transcription events (Murani et al., 2009; Berulava and Horsthemke, 2010); however none of MACROD2 SNPs reported so far fall within the first intron. At the time of submission of this manuscript, we have interrogated the Catalogue Of Somatic Mutations In Cancer (COSMIC) and retrieved 94 SNPs hitting MACROD2 in various cancers. COSMIC is the world’s largest and most comprehensive resource for exploring the impact of somatic mutations in human cancers, offered by the Wellcome Sanger Institute^2^. Additionally, it includes a catalog of genes with mutations that are causally implicated in cancer. Our bioinformatics analysis on SNPs frequencies of the interested genomic regions and functional consequences of COSMIC variants hitting the MACROD2 gene strikingly uncovered that 82 out of 94 SNPs involve exons and that most of these variants were synonymous (18%) or non-synonymous (58%) amino acid substitutions (Figure 4). This could suggest that, at least for human cancers, the vast majority of reported SNPs might affect MACROD2 protein function and stability, and not non-coding regulatory regions within its gene.

Nevertheless, the mouse genetics and phenotyping data presented here argue against an independent or sufficient role of MACROD2 gene in response to metabolic and tumorigenic stresses, in absence of epistatic interactions or of a susceptible genetic background.

## Author Contributions

MV designed and directed the study and drafted the paper. OLR and MV performed the experiments. TM performed the bioinformatic analyses. All authors edited and approved the final version.

## Conflict of Interest Statement

The authors declare that the research was conducted in the absence of any commercial or financial relationships that could be construed as a potential conflict of interest.

## Footnotes

1 http://annovar.openbioinformatics.org/

2 https://www.sanger.ac.uk/science/tools/cosmic
